# The role of non-*Helicobacter* *pylori* bacteria in the pathogenesis of gastroduodenal diseases

**DOI:** 10.1186/s13099-022-00494-0

**Published:** 2022-05-23

**Authors:** Langgeng Agung Waskito, Yudith Annisa Ayu Rezkitha, Ratha-korn Vilaichone, Titong Sugihartono, Syifa Mustika, I Dewa Nyoman Wibawa, Yoshio Yamaoka, Muhammad Miftahussurur

**Affiliations:** 1 Department of Internal Medicine, Faculty of Medicine, Universitas Airlangga, Surabaya, Indonesia; 2 Helicobacter pylori and Microbiota Study Group, Institute of Tropical Disease, Universitas Airlangga, Surabaya, Indonesia; 3 Faculty of Medicine, Universitas Muhammadiyah Surabaya, Surabaya, Indonesia; 4 Gastroenterology Unit, Department of Medicine, Faculty of Medicine, Thammasat University Hospital, Pathumthani, Thailand; 5 Digestive Diseases Research Center, Thammasat University, Pathumthani, Thailand; 6 Department of Medicine, Chulabhorn International College of Medicine at Thammasat University, Pathumthani, Thailand; 7 Division of Gastroentero-Hepatology, Department of Internal Medicine, Faculty of Medicine, Dr. Soetomo Teaching Hospital, Universitas Airlangga, Jalan Mayjend Prof. Dr. Moestopo No. 6-8, Surabaya, 60286 Indonesia; 8 Division of Gastroentero-hepatology, Department of Internal Medicine, Dr. Saiful Anwar General Hospital, Malang, Indonesia; 9 Division of Gastroentero-Hepatology, Department of Internal Medicine, Sanglah General Hospital, Faculty of Medicine, Udayana University, Denpasar, Indonesia; 10 Department of Environmental and Preventive Medicine, Oita University Faculty of Medicine, 1–1 Idaigaoka, Hasama-machi, Yufu-City, Oita 879-5593 Japan; 11 Department of Medicine, Gastroenterology and Hepatology Section, Baylor College of Medicine, Houston, TX USA

**Keywords:** Gastric microbiota, *Helicobacter pylori*, Cancer, Gastric cancer, Gastritis, Precancerous lesion

## Abstract

Over the past decade, the development of next-generation sequencing for human microbiota has led to remarkable discoveries. The characterization of gastric microbiota has enabled the examination of genera associated with several diseases, including gastritis, precancerous lesions, and gastric cancer. *Helicobacter pylori* (*H. pylori*) is well known to cause gastric dysbiosis by reducing diversity, because this bacterium is the predominant bacterium. However, as the diseases developed into more severe stages, such as atrophic gastritis, premalignant lesion, and gastric adenocarcinoma, the dominance of *H. pylori* began to be displaced by other bacteria, including *Streptococcus*, *Prevotella*, *Achromobacter*, *Citrobacter*, *Clostridium*, *Rhodococcus*, *Lactobacillus*, and *Phyllobacterium*. Moreover, a massive reduction in *H. pylori* in cancer sites was observed as compared with noncancer tissue in the same individual. In addition, several cases of *H. pylori*-negative gastritis were found. Among these individuals, there was an enrichment of *Paludibacter*, *Dialister*, *Streptococcus*, *Haemophilus parainfluenzae*, and *Treponema*. These remarkable findings suggest the major role of gastric microbiota in the development of gastroduodenal diseases and led us to the hypothesis that *H. pylori* might not be the only gastric pathogen. The gastric microbiota point of view of disease development should lead to a more comprehensive consideration of this relationship.

## Background

Gastrointestinal (GI) diseases have caused an increasing burden, with more than 80 million deaths worldwide. Diarrheal diseases and cirrhosis are among the top 10 death-causing gastroduodenal diseases in developing and middle- to low-income countries [1]. On the other hand, in developed and high-income countries, GI malignancies are one of the death-causing diseases, of which colon, liver, and gastric cancers are the most prevalent [2]. Gastric cancer is among the five most common digestive cancers worldwide, along with colorectal, pancreatic, and esophageal cancers. Altogether, these cancers are responsible for the deaths of > 365,000 people per year in Europe, accounting for almost one in every three cancer-related deaths [3]. In addition, patients with advanced gastric cancer in Europe had a 5-year survival rate of < 30%. In 2018, the estimated age-standardized incidence of gastric cancer was 15.7 per 100,000 male population and 7.0 per 100,000 female populations worldwide [4]. More than 90% of all gastric cancers are gastric tissue adenocarcinoma, and the remaining are lymphomas or gastric malignancies of the GI stromal tissue [5]. Several factors highly influence the development of these gastroduodenal diseases, including host genetic polymorphisms related to vulnerability and environmental factors associated with diets, lifestyle habits, and infection pathogens, especially *Helicobacter pylori* (*H. pylori*).

*H. pylori* is believed to cause several gastroduodenal diseases, including chronic gastritis, peptic ulcer diseases, gastric adenocarcinoma, and mucosa-associated lymphoid tissue lymphoma [6, 7]. *H. pylori* is estimated to have infected 4.4 billion people worldwide in the general adult population from 1970 to 2016, with the highest incidence in Africa (79.1%), Latin America and the Caribbean (63.4%), and Asia (54.7%) and a lower incidence in Northern America (37.1%) and Oceania (24.4%) [8]. Although it was previously reported that only 1–2% of patients with *H. pylori* infection developed gastric cancer in Japan and Taiwan [9], the recent consensus and a meta-analysis reported that *H. pylori* eradication could reduce the incidence of gastric cancer by 0.55-fold (95% confidence interval [CI], 0.42–0.72) [10]. These findings suggest that *H. pylori* infection still plays a major role in the development of gastric cancer; thus, eradication therapy for *H. pylori* infection is an effective approach to reducing the burden of gastric cancer.

Although *H. pylori* infection is highly associated with gastroduodenal diseases, several studies have reported the prevalence of gastritis in the absence of *H. pylori* infection [11, 12]. Even in more severe conditions such as premalignant or gastric adenocarcinoma, a low abundance of *H. pylori* was reported [13]. In addition, there has been an increase in the sensitivity of current methods for detecting specific bacterial communities in the microenvironment, and current computational biology can predict the taxonomy associated with certain diseases [14]. These findings have brought about the possibility of discovering other agents responsible for the development of gastroduodenal diseases in conjunction with *H. pylori* infection. In this review, we discuss the currently known role of gastric microbiota in the development of gastroduodenal diseases, which suggests that *H. pylori* is not the only agent for the development of gastroduodenal diseases.

### Gastric bacterial microbiome profile

The human stomach is a special area in the human GI organ system. It has a unique bacterial community resulting from a combination of gastric acid secretion, mucus thickness, and peristaltic movements [15]. With that combination of gastric physiology, the gastric cavity was believed to be a sterile environment because of its high acidity, which is unsuitable for bacterial colonization [16]; however, several acid-resistant bacteria could live in the stomach mucosa and are derived from the transient bacteria in the mouth and food, including *Streptococcus*, *Neisseria*, and *Lactobacillus*, with concentrations of approximately < 10^3^ colony-forming units/mL [17]. Furthermore, the discovery of *H. pylori* in 1983 opened the era of the pathogen responsible for gastroduodenal diseases [18].

In recent years, as a result of the introduction of the bacterial 16S rDNA identification technique, molecular technology has undergone rapid development. This approach may prove the existence of a gastric microbial community without the use of any culture technique. Gastric mucosal-associated microbes such as *Enterococcus*, *Pseudomonas*, *Staphylococcus*, and *Stomatococcus* were discovered in the early phase of molecular method studies [19]. A study conducted in the United States that identified gastric microbial communities in patients with gastric disease found 128 kinds of phylotypes belonging to five major phyla of Proteobacteria, Firmicutes, Bacteroidetes, Actinobacteria, and Fusobacteria with 1506 types of non-*H. pylori* bacteria [20]. Another study conducted in Hong Kong with similar gastric conditions showed identification of 1223 non-*H*. *pylori* bacteria that could be classified into 133 kinds of phylotypes belonging to eight bacterial phyla [21]. Those studies were conducted in America and Hong Kong but yielded similar bacterial phyla, with five of eight identified phyla in the latter (*Proteobacteria*, *Firmicutes*, *Bacteroidetes*, *Actinobacteria*, and *Fusobacteria*) the same between different populations. These data suggest the similarity of gastric microbial communities observed from distinct populations. Another study conducted pyrosequencing analyses of gastric mucosa-associated bacteria in six healthy subjects and obtained 262 phylotypes belonging to 13 classes, including some that had not been confirmed by other studies, such as *Chlamydia* and *Cyanobacteria* [22]. In general, the human stomach holds a core microbiome. Although the gastric microbiome is highly variable between individuals, recent studies have detected five major phyla in the stomach, including Firmicutes, Bacteroidetes, Actinobacteria, Fusobacteria, and Proteobacteria. The predominant genera in the stomach are *Prevotella*, *Streptococcus*, *Veillonella*, *Rothia*, and *Haemophilus* [23]. However, when interpreting the current findings on the gastric “core microbiome,” caution is necessary, as these findings might be obtained from sequence-based techniques with only limited data on bacterial viability. To confirm the viability of the discovered microbes, further study is necessary.

There are several factors that affect the variability of gastric microbiota, including diet and supplementary nutrient intake, geographic origin, aging, medication (e.g., antibiotics, proton pump inhibitors [PPIs], and H2 antagonists), *H. pylori* infection, and other systemic diseases [24–27]. The variability in gastric microbial composition could be a normal variation or lead to a dysbiosis. Basically, a dysbiosis is defined as the microbial imbalance in a certain microenvironment [28]. A dysbiosis is usually associated with a certain disorder—either local organ or systemic manifestation. Whether dysbiosis is caused by the disorder (e.g., *H. pylori* infection, cancers, or autoimmune diseases) or is causing the disorder (e.g., vulnerability to infection, chronic metabolic diseases, and cancers) remains unclear. Accumulated evidence supports the hypothesis that gastric dysbiosis is associated with the development of gastroduodenal diseases [29–31]. These findings led to a new perspective on the gastric microbial environment as a whole system responsible for the pathogenesis of disease.

### *H. pylori* as standalone pathogen and its interaction with gastric microbiota

*H. pylori* is widely known to be a major risk factor for the development of various gastroduodenal diseases, including chronic gastritis, ulcers, and gastric cancer. *H. pylori* has been classified as a class I carcinogen by the International Agency for Research on Cancer [32, 33] because of the close relationship between *H. pylori* infection and the incidence of gastric cancer. Worldwide, *H. pylori* has been associated with at least 90% of all noncardia gastric cancer cases [34]. Based on geographical distribution, a major overlap was observed between *H. pylori* positivity and the incidence of gastric cancer in various countries worldwide. Because of this major overlap in distribution and the classification of *H. pylori* as a class I carcinogenic factor, several studies have demonstrated a causal relationship between the presence of *H. pylori* and the gastric cancer development. A systematic review of 12 studies showed that the prevalence of *H. pylori* infection in noncardia gastric adenocarcinoma was threefold higher (95% CI, 2.3–3.8) than that in noninfected individuals. However, when the pooled analysis was restricted to 10 or more years after the diagnosis of *H. pylori* infection, the prevalence increased by 5.9-fold (95% CI, 3.4–10.3) [35]. These findings provide evidence that links *H. pylori* infection to gastric cancer.

There is a well-recognized association between *H. pylori* infection and the incidence of gastric cancer. However, the actual pathogenic pathway of *H. pylori*-inducing gastric cancer has not been completely elucidated with clear evidence of *H. pylori-*inducing DNA damage and inflammation [36, 37]. Numerous factors affect the development of various diseases after the colonization of *H. pylori* in the human stomach, including host genetic susceptibility, *H. pylori* virulence factors, and individual lifestyle and dietary habits. Because to its polymorphism, the genetic susceptibility of the hostis involved in the gastroduodenal disease development and increases the risk of gastric cancer. Many genetic polymorphisms have been reported to be significantly associated with the development of gastric cancer. However, among the best studied are those that encode interleukin (IL)-1β, IL-1 receptor antagonist, anti-inflammatory IL-10, tumor necrosis factor (TNF)–α proinflammatory cytokines, and the IL-17 cytokine family. The association of genetic variability in the promoters or noncoding regions of these genes with increased risk for the development of gastric cancer has been well documented [38–41]. In addition, several gene polymorphisms were reported to be highly associated with the development of atrophic gastritis, such as transforming growth factor-β1, TNF-α, interferon-γ, and IL-6 in *H. pylori*-negative individuals [42]. In addition to genetic susceptibility, dietary patterns that include a high intake of salt and smoking habits have been reported to increase the odds for the development of gastroduodenal diseases, including gastric cancer. Besides affecting an individual’s susceptibility to gastric cancer, the host’s genetic and habitual routine factors also affect the gastric microbial community. In a study comparing the gastric microbiomes between Indian, USA, Chinese, and Colombian populations, a distinction was observed that separated into three cluster populations, consisting of samples from United States and Colombia, which were formed closely with each other; the Indian samples; and the Chinese samples [43]. In addition, two different populations with distinct risks of gastric cancer in Colombia showed different microbial communities [26]. In Indonesia, which is a large country with various ethnicities, also showed a significantly different gastric microbiome, which might be responsible for the increase in the odds for developing *H. pylori* infection [27]. When the gastric microbiomes of twins were compared, genetic alteration of gastric microbiota showed no role in the difference. In that study, no signs of increasing coexisting bacterial communities were found in twins when compared with an unrelated person of the same ethnicity [44]. These findings emphasize that the host and population can affect the gastric microbial community via the design of its own core microbiome in each population.

Among the *H. pylori* virulence factors, CagA is the most documented as associated with disease pathogenesis. It is encoded as part of the cag pathogenicity island, a type IV secretion system playing the role of a syringe that facilitates CagA protein entrance into host cells [45]. In general, a person infected by *H. pylori* containing CagA will develop greater gastric damage, including gastritis (superficial and atrophic), duodenal ulcers, and gastric carcinogenesis [46]. CagA mainly affects the induction of more severe clinical outcomes via several mechanisms, including a reduction in glycogen synthase kinase–3 activity, failure to maintain organ structure, activation of the ERK pathway, change in cellular polarity, alteration of cell cycles, promotion of cell proliferation, and replacement of gastric epithelial cells into intestine-specific cells [47]. Alongside CagA, another important virulence factor is VacA, which encodes vacuolating cytotoxin and plays a vital role in the survival of *H. pylori* by inducing the flow of ions and nutrients, altering the integrity of the gastric epithelium [48]. This gene has variable genetic characteristics in several regions, which could be used to stratify the levels of *H. pylori* virulence [47–49]. In addition, numerous outer membrane proteins were significantly associated with *H. pylori* virulence. Recent findings showed that *Helicobacter* outer membrane protein Q (HopQ) interacts with the carcinoembryonic antigen-related cell adhesion molecule family and enhances the adherence of *H. pylori* to gastric mucosal cells. In addition to its function of promoting adherence to the host cell, HopQ is also a dependent factor of the T4SS translocating CagA protein in the host cell [50]. The virulence of *H. pylori* is important not only in the development of mucosal inflammation but also in the alteration of the gastric microbial community. An experiment in an animal model revealed that even though *H. pylori* in gerbils infected by *cagA* isogenic mutant had a diversity similar to that in the wild type, its composition was different, suggesting the ability *H. pylori* to change the microbial community in a *cagA*-dependent manner [51]. These findings suggest that *H. pylori* strain-specific virulence genes not only affect its ability to colonize, causing mucosal damage and inducing the secretion of several proinflammatory cytokines, but also altered the gastric microbiota. Considering the many important virulence factors of *H. pylori*, which other virulence factor is important to the alteration of the gastric microbial community should be identified.

Although *H. pylori* has been well documented as being closely related to gastroduodenal diseases, not all infected individuals develop cancer or even ulcers. Most cases are gastritis. A recent animal model study investigated whether malignant lesions in rodents actually represented cancer. The lesions were reported as putative malignant lesions instead of proliferative metaplastic or reactive lesions. In addition, experiments conducted with organoids constructed from gastric cancer mouse models failed to induce tumors in a xenograft model, whereas the controls produced tumors [52]. These findings confirmed the complexity of gastric cancer development, which can be related to a specific human–*H. pylori* genetic mechanism, the possible roles of certain gastric microbial profiles, and many other factors. Although studies are still in the early phase, factors other than non-H. *pylori* bacteria may be responsible for the development of gastroduodenal diseases.

### *H. pylori*-negative gastritis and its microbial community

Gastritis is defined as inflammation that occurs in the gastric mucosa. It is most commonly observed in the spectrum of gastroduodenal diseases. Histologically, gastritis is divided into two categories, namely, superficial gastritis (nonatrophic) and atrophic gastritis [53]. Superficial gastritis is defined as an inflammation of the gastric mucosa and is evaluated based on the appearance of polymorphonuclear infiltration in acute gastritis and mononuclear infiltration in chronic gastritis. On the other hand, atrophic gastritis is defined as loss of the appropriate glands [54]. Several etiological factors lead to gastritis, including chemical agents (e.g., nonsteroidal anti-inflammatory drugs, dietary factors, alcohol, and bile reflux), physical agents (e.g., radiation), immune-mediated conditions, and infections (e.g., *H. pylori*, parasites, and viruses) [53]. The most common etiological factor is *H. pylori* infection. Gastritis the results from *H. pylori* infection is often chronic, with some cases progressing to atrophic gastritis.

Although in clinical practice, *H. pylori* has been widely accepted as causing most or all cases of gastritis, some patients still have *H. pylori*-negative gastritis. This category might be slightly difficult to define because of some limitations in the detection of *H. pylori* infection from widely available diagnostic modalities. After considering the use of several screening methods for *H. pylori*, the prevalence of *H. pylori*-negative gastritis was found in one study to be approximately 21% in the United States [55]. The authors of that study reported that several differential diagnoses could explain their findings, but the observed gastritis was mostly more focal and milder than *H. pylori* gastritis and tended to be chronic rather than chronic–active or active. Thus, the etiology of the observed gastritis was not clearly determined. In addition, using a similar approach, *H. pylori*-negative gastritis was also observed in approximately 27% of all cases of gastritis in Indonesia [11]. Because this phenomenon is certainly caused by an agent, the gastric microbiota approach might provide some insight into the associated agent.

Knowing that the 16s rRNA sequence approach will yield more sensitive results, several studies have described the microbial community in patients with gastritis without *H. pylori* infection. A study conducted in Mongolia consisting of 11 patients with *H. pylori*-negative gastritis revealed a similar diversity index between these patients and individuals with normal mucosa. With regard to the gastric microbial composition, the relative abundance in the *H. pylori*-negative group showed a decreased amount Proteobacteria and increments in the Bacteroidetes population with the introduction of Spirochaetes as compared with the healthy group, in which the proportions of Proteobacteria, Bacteroidetes, and Firmicutes were evenly distributed [56]. Screening for *H. pylori* noninfection was based on the relative abundance of 2%, which is typically found in *H. pylori*-negative individuals [57, 58]. By applying similar criteria, a study in Indonesia also reported that *Paludibacter* sp. bacteria increased the abundance in the *H. pylori*-negative gastritis patients [27]. These findings suggest that, even in the absence of *H. pylori*, it is still possible to detect typical gastritis caused by infection and that the gastric microbiota was also altered. Indeed, recent studies describing the microbiota and gastritis especially in the absence of *H. pylori* are still limited to a cross-sectional design, which still provides two-way hypotheses. Therefore, studies using a more causative design, such as cohort studies, animal studies, or in vitro models, are needed to confirm the role of gastric dysbiosis in the pathogenesis of gastritis.

### Lack of *H. pylori* in premalignancy and adenocarcinoma

Determination of *H. pylori* infection in clinical practice was based on several diagnostic modalities, including the visualization of *H. pylori*-like bacteria (spiral shape) from the gastric biopsy, the appearance of *H. pylori* antibody from enzyme-linked immunosorbent assay, and detection of *H. pylori* antigen from the stool and/or from urease-based tests [59]. When tested on individuals with gastritis, these diagnostic methodologies have excellent performance, but they are widely reported to have a very low *H. pylori* infection positivity rate among patients with gastric cancer or in premalignant patients. The positivity rate was even lower than in patients with gastritis and ulcer diseases [60]. Because of the confidence that *H. pylori* must exist, the most common explanation in those situations relies on the possible “false-negative” result [59–61].

Compared with other diagnostic modalities, the sequence of the 16s rRNA approach showed greater sensitivity. The low prevalence of *H. pylori* as detected by conventional methodologies is probably due to the dysbiosis caused by the development of disease. After applying *H. pylori* detection using the next-generation sequencing approach, the lower abundance of *H. pylori* among gastric cancer and premalignant individuals was reinforced. Among the *H. pylori*–positive individuals, *H. pylori* was the most predominant bacteria in the benign condition, such as gastritis and ulcer. However, when it was developed as a premalignancy (e.g., atrophic gastritis and intestinal metaplasia) and gastric cancer, the dysbiosis began to occur, and a large amount of other bacteria colonized the gastric mucosa [13, 62]. One study in Portugal showed that although that individuals with gastric cancer had lower diversity than individuals with chronic gastritis did, the abundance of *H. pylori* was reduced significantly and was replaced by non-*H*. *pylori* Proteobacteria [29]. In addition, among *H. pylori*-infected individuals with gastric cancer, *H. pylori* still maintained its dominance; however, it was reduced significantly as the disease progressed to gastric cancer, while the diversity increased [63, 64]. Interestingly, the gastric cancer lesion did not cover the entire gastric cavity, and it is also interesting to observe the different microbial profiles between cancer and normal specimens within the same individual. The gastric normal location showed the highest observed OTU compared with the peri-tumor lesion and the tumor lesion. Although *H. pylori* still showed the highest abundance across those three locations, it was significantly reduced in the tumor lesion compared with the normal lesion [65]. These results suggest that even though the dysbiosis resulting from disease development led to either a higher or lower diversity index, the abundance of *H. pylori* was severely reduced, suggesting that the ability of *H. pylori* to colonize was massively reduced by the arrival of other bacteria, which could allow it to easily stay in more favorable conditions and might promote more severe disease development.

In addition to being affected by external factors, such as lower acidity as well as the attack of other bacteria, the lower abundance of *H. pylori* is also affected by the *H. pylori* activity itself. The activity of *H. pylori* is dependent on its shape, which is known to be spiral or coccoid form. This coccoid form is an inactive state of *H. pylori* that is affected by several factors, including antibiotic exposure, extreme pH change, and a low amount of metabolic substances [66]. The production of *H. pylori* urease capability is increased when it lives in a highly acidic environment, a condition that is absent in both cancer and the precancerous state (atrophic gastritis and intestinal metaplasia). This acidic condition allows *H. pylori* to produce urease and live in the most active and optimal form. When in the less acidic condition, *H. pylori* adapts and changes its form into a coccoid shape, forming a biofilm [67]. In this state, *H. pylori* still exists; however, is does not colonize as actively and its biological function is highly reduced. These factors may preclude its identification by several conventional tests. However, there remains a lack of knowledge regarding whether *H. pylori*, after assuming its coccoid form, could change back into a spiral shape and recover its virulence ability. Further studies determining the factors and mechanisms related to the reversion into a spiral shape and its maximum virulence potential would be of interest.

### Are the new candidates the real villain?

The development of gastroduodenal diseases, including gastritis, ulcers, and gastric cancer, is complex. Infection-related gastritis might be involved only in inflammation caused by pathogenic aggression. With regard to ulcers and gastric cancer, the mechanism begins as a complex interaction between host, agent, and environmental factors. Currently, the well-accepted concept of gastric cancer pathogenesis is Correa’s pathway, with confounding factors such as high-salt diets and other carcinogenic substances that promote the carcinogenic pathway [68]. However, investigation of the microbiome in cancer research and findings regarding dysbiosis related to cancer pathogenesis open opportunities for other factors, which are, in this case, other bacterial agents of cancer development.

Studies that identified microbial candidates related to gastritis have mostly included precancerous or gastric cancer conditions. Because it is the mildest disease in the disease spectrum, gastritis was primarily regarded as the control group. An investigation revealed that the dysbiosis related to the incidence of gastritis was mostly caused by *H. pylori*, because the pathogen was the most abundant and dominant taxon in patients with gastritis [13]. When limited to patients with *H. pylori* gastritis only, the associated dysbiosis was slightly different. A study in Indonesia that determined the association between non-H. *pylori* bacteria and gastritis cases showed that the abundance of *Paludibacter* and *Dialister* species was significantly increased in infected patients as compared with individuals with healthy gastric mucosa [27]. In addition, the Mongolian population, which has a high incidence of gastric cancer, showed augmentation of dysbiosis by the *Streptococcus*, *Haemophilus parainfluenzae*, and *Treponema* taxa in patients with *H. pylori*-negative gastritis [56]. These findings suggest potential new candidate pathogens that might be related to the development of gastritis in the absence of *H. pylori* infection.

**Fig. 1:**
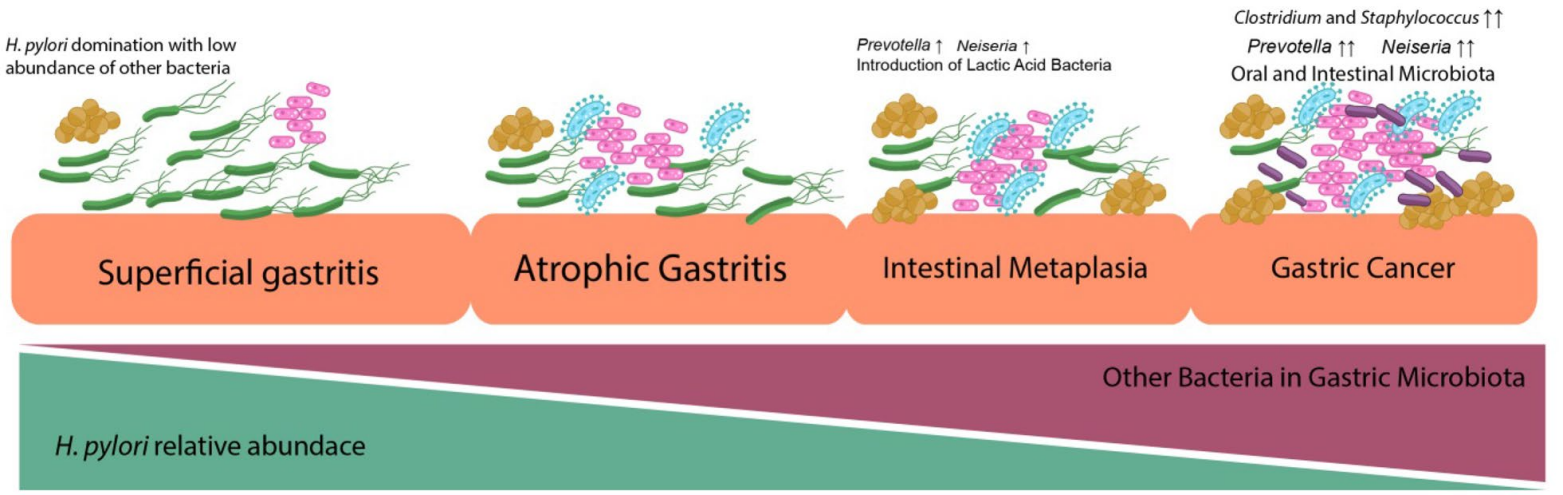
Association of *Helicobacter pylori* abundance with the different stages of gastric conditions. The presence of *H. pylori* was dominant in the superficial gastritis condition; thus, this domination reduced microbial diversity. In atrophic gastritis and intestinal metaplasia, the relative abundance of *H. pylori* began to decrease with the introduction of other bacteria, including the incremental of *Prevotella* sp. and *Neisseria* sp. In the gastric cancer condition, *H. pylori* started to deteriorate with a significantly increased amount other bacteria, including oral cavity microbiota, intestinal microbiota, and lactic acid bacteria

Bacterial candidates related to the development of gastric cancer have been investigated over the past few years in several populations, with intriguing results. In general, the dysbiosis characteristics of gastric microbiota can be used to distinguish gastric cancer from other diseases. Even though gastric dysbiosis occurred with inconsistent shifting diversity index values, a decrease in the relative abundance of *H. pylori* and incremental changes in the relative abundance of other bacteria have been frequently reported [29, 62, 69] (Fig. 1). Table 1 summarizes the information known regarding gastric dysbiosis associated with gastroduodenal diseases. In gastric cardia adenocarcinoma, the observed microbial community was mainly composed of Firmicutes, Bacteroidetes, and Proteobacteria at the phylum level. At the genus level, an increase in relative abundance was reported in *Prevotella*, *Streptococcus*, *Veillonella*, *Haemophilus*, and *Neisseria* [70]. In addition, a significant difference in gastric microbial community was also observed between nonatrophic gastritis and gastric cancer, in which the diversity index was gradually reduced when diseases progressed from gastritis to gastric cancer, with increased abundance of non-H. *pylori* proteobacteria. In a validation cohort analysis, the populations of several bacteria, including *Streptococcus*, *Prevotella*, *Achromobacter*, *Citrobacter*, *Clostridium*, *Rhodococcus*, *Lactobacillus*, and *Phyllobacterium*, were significantly increased in patients with gastric cancer as compared with those with chronic gastritis [29]. At the species level, populations of *Prevotella melaninogenica, Streptococcus anginosus*, and *Propionibacterium acne* were increased in tumor tissues, whereas those of *H. pylori* and *Bacteroides uniformis* were deceased [65]. In addition, an analysis of gastric microbial communities from different stages of gastric cancer development revealed the importance of *Peptostreptococcus stomatis*, *S. anginosus*, *Parvimonas micra*, *Slackia exigua*, and *Dialister pneumosintes* in the progression of gastric cancer, as they were found to coexist from the precancerous stage [30].

**Table 1:** Characteristics of gastric dysbiosis in different gastroduodenal diseases

Samples and subjects	Characteristics of dysbiosis	References
5 Dyspepsia and 10 gastric cancer samples	Domination of different species of the genera *Streptococcus*, *Lactobacillus*, *Veillonella*, and *Prevotella* with low abundance of *H. pylori* among patients with gastric cancer.	[86]
5 Patients with nonatrophic gastritis, intestinal metaplasia, and intestinal-type gastric cancer	Bacterial diversity index ranged from 8 to 57, with a decrease from atrophic gastritis to intestinal metaplasia and gastric cancer. Significant decrease in bacterial diversity observed from gastric cancer compared with nonatrophic gastritis.	[87]
10 Patients with chronic gastritis, 11 patients with noncardia gastric cancer, and 10 patients with intestinal metaplasia	Increased relative abundance of *Streptococcaceae* family with lower relative abundance of *Helicobacteraceae* family among the gastric cancer group compared with the chronic gastritis and intestinal metaplasia groups.	[64]
212 Patients with chronic gastritis and 103 patients with gastric cancer enrolled with only 12 patients (6 cancer and 6 chronic gastritis) carried out for microbiome analysis	Five genera bacteria, including *Lactobacillus*, *Escherichia Shigella*, *Nitrospirae*, *Burkholderia fungorum*, and *Lachnospiraceae*, were enriched among patients with gastric cancer. The presence of *H. pylori* heavily changed the structure of the microbiota with a small influence on the relative proportion of other bacteria.	[88]
81 Patients with chronic gastritis and 54 patients with gastric cancer	In general, the gastric carcinoma microbiota was characterized as having reduced microbial diversity with a decreased abundance of *Helicobacter* and enrichment of other bacteria genera, mostly represented by intestinal commensal bacteria. Specifically, *Citrobacter*, *Lactobacillus, Clostridium*, and *Rhodococcus* were also significantly more abundant in gastric carcinoma. *Helicobacter*, *Neisseria*, *Prevotella*, and *Streptococcus* were most abundant in the microbiota of patients with chronic gastritis.	[29]
21 Patients with superficial gastritis, 23 patients with atrophic gastritis, 17 patients with intestinal metaplasia, and 20 patients with gastric cancer	In general, there was significant mucosa microbial dysbiosis in the intestinal metaplasia and gastric cancer cases compared with cases of superficial gastritis. Several species, including *Peptostreptococcus stomatis*, *Streptococcus anginosus*, *Parvimonas micra*, *Slackia exigua*, and *Dialister pneumosintes*, were centralites in the ecological network analysis.	[30]
110 *H. pylori*-negative individuals and 7 *H. pylori*–positive patients	Significantly lower diversity among *H. pylori*–positive patients. A high abundance of *Paludibacter* sp. and *Dialister* sp. was observed among individuals with gastric damage.	[27]
24 Controls negative for *H. pylori* and with nongastritis with 11 additional *H. pylori*-negative gastritis patients with 40 *H. pylori*–positive patients	Significantly increased abundance of *Streptococcus* sp. and *Haemophilus parainfluenzae* among *H. pylori*-negative gastritis patients, whereas *Treponema* sp. was uniquely found in *H. pylori*-negative gastritis patients based on occurrence.	[56]
120 Patients without cancer (20 normal, 20 gastritis, 40 atrophic gastritis, and 40 intestinal metaplasia) and 48 patients with gastric cancer	The least diversity was seen among the gastritis and atrophic gastritis group. *Lactobacilli* and *Enterococci* were the dominant genus in several patients with cancer, especially in the absence of *H pylori*. In addition, *Carnobacterium*, *Glutamicibacter*, *Paeniglutamicibacter*, *Fusobacterium*, and *Parvimonas* were associated with gastric cancer regardless of *H pylori* infection.	[89]
230 Normal tissues, 247 peritumoral tissues, and 229 tumoral tissues from 276 patients with gastric cancer	The tumor microhabitat showed an increased abundance of *Prevotella melaninogenica*, *Streptococcus anginosus*, and *Propionibacterium acne* with decreased abundance of *H. pylori*, *Prevotella copri*, and *Bacteroides uniformis.*	[65]
288 Controls and 268 patients with gastric cancer	There is a different description of microbial community from different levels. At the species level, the patients with gastric cancer had higher relative abundances of *H. pylori*, *Propionibacterium acnes*, and *Prevotella copri* than the controls did, whereas the relative abundance of *Lactococcus lactis* was higher in the healthy controls than in the patients.	[71]
36 Paired nontumor tissue and gastric cardia adenocarcinoma samples	Increased relative abundance of *Prevotella*, *Streptococcus*, *Veillonella*, *Haemophilus*, and *Neisseria* among carcinoma tissue compared with nontumor tissue specimens.	[70]

Gastric dysbiosis not only causes microbial imbalance in the gastric cavity but also leads to functional shifting that might be responsible for the development of gastroduodenal disease. A study conducted in South Korea showed that the associated bacteria in the gastric cancer population compared with controls were *H. pylori*, *Propionibacterium acnes*, and *Prevotella copri* [71]. The overabundance of *P. acnes* is associated with enhancement of gastric cancer development via the production of proinflammatory cytokines such as IL-15 [72]. It has been strongly suggested that *P. copri* induces inflammatory conditions that might also be responsible for gastric cancer development [73]. In addition, recent findings showed an increased abundance of lactic acid bacteria (LAB), including *Streptococcus* [30], *Lactobacillus* [29–31], *Bifidobacterium*, and *Lactococcus*, in patients with gastric cancer [74]. LAB have been considered to promote the development of gastric cancer via several mechanisms, including the production of *N*-nitroso compounds, reactive oxygen species, and anti-*H. pylori* properties [75]. The most powerful evidence proving the role of LAB in gastric cancer development was obtained from an insulin-gastrin (INS-GAS) transgenic mouse model. In that study, GI intraepithelial neoplasia, which is associated with a strong upregulation of proinflammatory and cancer-related genes, was promoted in male INS-GAS mice colonized with a specific microbiome (including *Lactobacillus* murinus ASF361, *Clostridium* ASF356, and Bacteroides ASF519) [76]. In addition, the evidence to date clearly shows that lactate, the metabolite of LABs, can promote inflammation, angiogenesis, and metastasis and regulate the immune response [77], which might influence the outcome of gastric cancer. These findings emphasize the involvement of non-H. *pylori* bacteria in the development of gastric cancer in some manner.

### The future of *H. pylori* and gastric microbiota

Undeniably, *H. pylori* is an important factor in the development of gastroduodenal diseases. Since its discovery in 1983, it has remarkably changed the perspective on gastroduodenal diseases, and eradication therapy could prevent the progression of gastric mucosal conditions and carcinogenesis [61–78]. However, because of the wide clinical spectrum of infected individuals, which ranges from superficial gastritis to adenocarcinoma, and from the *H. pylori* standpoint, different virulence characteristics of *H. pylori* may exist between the outcomes. The bacterial genome-wide association approach has shown promising outcomes of several single-nucleotide polymorphisms that were highly correlated with and increased the odds for gastric cancer [79]. Although the findings are limited to only one study, this approach described how *H. pylori* operates as a pathogen of different clinical outcomes. The application of this approach in high-risk gastric cancer populations, such as East Asian populations, is interesting.

An increasing amount of data on relationship between the involvement of gastric microbiota and its associated dysbiosis with the development of gastroduodenal diseases clearly show that *H. pylori* is not the single responsible pathogen. The gastric microbial community is undeniably involved in the disease pathogenesis via several mechanisms. However, the currently investigated gastric dysbiosis and even the discovered gastric microbial biomarkers are still limited to a two-way association with the disease. The have been no studies investigating whether transferring of the dysbiosis microbial community leads to diseases. Furthermore, no study has used the opposite approach to examine whether the restoration of the gastric microbiota from dysbiosis to an equilibrium state might improve the clinical condition. In addition, current findings are limited to the description of existing bacteria in a particular clinical condition and provide insufficient evidence or a proposed underlying mechanism of the pathogenesis. Therefore, there is a need for further studies examining the restoration of gastric dysbiosis while focusing on culture-omics and the possible mechanism of gastric microbiome-related gastroduodenal diseases.

In addition, a possible connection exists between gastric and gut microbiota in terms of development of disease and *H. pylori* infection. The long-term use of PPIs has been described to possibly disrupt the gut microbiota into a dysbiotic stage, with PPI being one of the main drugs for dyspepsia therapy and the *H. pylori* eradication regimen [80, 81]. In addition, one study described that patients with *H. pylori* infection had an increased diversity and richness of gut microbiota. A reduced number of Bacteroidetes and elevated numbers of Fimicutes and Proteobacter were observed in patients with gastritis as compared with healthy individuals [82]. A cohort study found that changes in gut microbiota in patients after radical distal gastrectomy resulted in an alteration of gut microbiota with increments of *Akkermansia* sp, *Esherichia*/*Shigella*, *Lactobacillus*, and *Dialister* [83]. The introduction of *Akkermansia* might be beneficial because the bacteria introduced in the gastric cancer group were depleted in an animal model experiment [84]. A biomarker discovery analysis yielded a combination of the genera *Lachnospira*, *Lactobacillus*, *Streptococcus*, *Veillonella*, and *Tyzzerella_3*, which showed promising performance in distinguishing patients with gastric cancer from healthy controls. This group of bacteria, specifically *Lactobacillus*, *Streptococcus*, and *Lachnospiraceae*, increased the number of CD3+T, CD4+T, and natural killer cells [85]. These findings confirmed the involvement of gut microbiota in gastric carcinogenesis. The management of gastroduodenal diseases should also take into consideration the alteration of gut microbiota.

## Conclusions

The involvement of *H. pylori* in the development of gastroduodenal diseases is an indisputable factor. However, recent findings on gastric microbiota in some spectrum diseases showed remarkably smaller populations of *H. pylori* and increments of other bacteria in gastric carcinogenesis, suggesting that *H. pylori* may not be the only pathogen responsible for the pathogenesis of the disease.

## Data Availability

Not applicable.
